# The doctor’s digital double: how warmth, competence, and animation promote adherence intention

**DOI:** 10.7717/peerj-cs.168

**Published:** 2018-11-12

**Authors:** Zhengyan Dai, Karl F. MacDorman

**Affiliations:** School of Informatics and Computing, Indiana University, Indianapolis, IN, USA

**Keywords:** Adherence, Anthropomorphism, Avatars, Computer animation, Doctor–patient simulations, Health literacy, Interactive narratives, Uncanny valley

## Abstract

**Background:**

Each year, patient nonadherence to treatment advice costs the US healthcare system more than $300 billion and results in 250,000 deaths. Developing virtual consultations to promote adherence could improve public health while cutting healthcare costs and usage. However, inconsistencies in the realism of computer-animated humans may cause them to appear eerie, a phenomenon termed the *uncanny valley*. Eeriness could reduce a virtual doctor’s credibility and patients’ adherence.

**Methods:**

In a 2 × 2 × 2 between-groups posttest-only experiment, 738 participants played the role of a patient in a hypothetical virtual consultation with a doctor. The consultation varied in the doctor’s Character (good or poor bedside manner), Outcome (received a fellowship or sued for malpractice), and Depiction (a recorded video of a real human actor or of his 3D computer-animated double). Character, Outcome, and Depiction were designed to manipulate the doctor’s level of warmth, competence, and realism, respectively.

**Results:**

Warmth and competence increased adherence intention and consultation enjoyment, but realism did not. On the contrary, the computer-animated doctor increased adherence intention and consultation enjoyment significantly more than the doctor portrayed by a human actor. We propose that enjoyment of the animated consultation caused the doctor to appear warmer and more real, compensating for his realism inconsistency. Expressed as a path model, this explanation fit the data.

**Discussion:**

The acceptance and effectiveness of the animation should encourage the development of virtual consultations, which have advantages over creating content with human actors including ease of scenario revision, internationalization, localization, personalization, and web distribution.

## Introduction

The persuasiveness of a message depends on its source, content, and the extent to which it is processed systematically or heuristically (Chaiken, 1980; Van Der Heide & Schumaker, 2013). Systematic processing assesses the content of a message, while heuristic processing assesses features unrelated to content. When the message source is another human, features affecting persuasion include physical appearance, perceived character traits, and behavior (Wood, Solomon & Englis, 2005). For character traits, the primary dimension of interpersonal perception is *warmth*, and the secondary dimension is *competence* (Cuddy, Fiske & Glick, 2008; Fiske et al., 2002). Warmth indicates the intention to help or harm others and competence indicates the capacity to do so (Fiske, Cuddy & Glick, 2007). Competence and facets of warmth, like *goodwill* and *trustworthiness*, have been used extensively to assess source credibility (McCroskey & Teven, 1999; McGinnies & Ward, 1980); they are important in the literature on persuasion because credible sources are more persuasive (Jain & Posavac, 2001; Maddux & Rogers, 1980; Pornpitakpan, 2004; Sertoglu, Catl & Korkmaz, 2014).

Persuasive strategies that increase perceived warmth and competence have been investigated in doctor–patient consultations (Cousin, Mast & Jaunin-Stalder, 2013; Janssen & Lagro-Janssen, 2012). Patients’ perception of their doctor’s warmth and competence increases *adherence*, that is, clinically unsupervised compliance with treatment advice. Communication strategies that express warmth increase patients’ perception of their doctor as caring, understanding, and empathy; thus, patients prefer these strategies (Cousin et al., 2012; Mast, Hall & Roter, 2007; O’Hair, 1986; O’Hair et al., 1987). The perception of a doctor’s empathy and competence (which includes expertise) increases the patients’ satisfaction with and adherence to treatment advice (Kim, Kaplowitz & Johnston, 2004; Ohana & Mash, 2015; Willson & McNamara, 1982). While satisfaction with communication increases adherence, dissatisfaction has the opposite effect (Burgoon et al., 1987; Marple et al., 2007). Warmth and competence improve the patents’ prognosis and quality of life by increasing adherence (Bennett et al., 2011; Borel et al., 2014; Swain et al., 2015).

Warmth, competence, attractiveness, and other factors affecting source credibility apply to virtual as well as real humans (Garau et al., 2005; Keeling, McGoldrick & Beatty, 2010). Virtual humans offer a familiar interface that can be used to change people’s beliefs, attitudes, intentions, and behaviors (Jin & Sung, 2010; Peña & Yoo, 2014) while disseminating professional advice. Along with the content of a message, virtual humans deliver nonverbal cues about warmth and competence (An et al., 2013; Anam, Andrade & Ruiz, 2016; Tongpeth, Du & Clark, 2018). These cues reduce the receiver’s uncertainty during communication and support decision-making (Burgoon & Hale, 1988; Burgoon & Walther, 1990). A virtual human’s nonverbal cues enable the experience of social presence, relatedness, and affinity (Bente et al., 2008)—an experience that precedes interpersonal trust (Cyr et al., 2007).

Although both real and virtual humans vary in form, behavior, and interactivity, virtual humans also vary in *human realism* (Bailenson et al., 2006). Designing perfectly or even consistently realistic virtual humans is an unsolved challenge (Chattopadhyay & MacDorman, 2016; Gilbert & Forney, 2015). We assume that all present-day virtual humans are realism inconsistent because the goal of realism is human indistinguishability, and its degree of attainment will vary depending on the difficulty of making each feature indistinguishable. Features that have been experimentally contrasted in realism include face and eyes (MacDorman et al., 2009; Seyama & Nagayama, 2007) and voice and body (Mitchell et al., 2011). A literature review concluded features that are inconsistent in their realism elicit feelings of *eeriness* (Kätsyri et al., 2015), a phenomenon known as the *uncanny valley* (Mathur & Reichling, 2016; Moore, 2012; Mori 2012). Eeriness could function like other negative evaluations to reduce source credibility.

Thus, a fundamental problem with virtual humans is that while delivering intended warmth and competence cues, they also deliver unintended cues about inconsistent realism (Chattopadhyay & MacDorman, 2016). These unintended cues may negate the positive effects of warmth and competence cues on persuasion. Specifically, a virtual doctor’s realism and potential for eeriness might affect a patient’s intention to adhere to treatment advice by influencing the virtual doctor’s persuasiveness relative to a real human. As this topic has not been studied empirically, the present study is intended to fill this gap.

Despite their differences, a virtual human could be as persuasive as a real human (Patel & MacDorman, 2015; Zanbaka, Goolkasian & Hodges, 2006)—or even more persuasive (Bickmore, Pfeifer & Paasche-Orlow, 2009). However, Raij et al. (2007) found that a virtual patient induced less empathy and rapport-building and worse attitudes in doctors than a real patient. Although these studies investigated the effect of realism on persuasion, only one employed an experimentally controlled stimulus, that is, a digital double (Patel & MacDorman, 2015). Moreover, the ways in which warmth, competence, realism, and eeriness together affect adherence intention in a hypothetical virtual consultation have not been investigated.

Based on the finding that warmth, including its goodwill and trustworthiness facets, and competence increase a source’s persuasiveness in both real and virtual environments, we hypothesized that (H1) *a high-warmth source will be more persuasive than a low-warmth source* and (H2) *a high-competence source will be more persuasive than a low-competence source.* In the virtual consultation, the high-warmth source was portrayed as a doctor with good bedside manner and the low-warmth source was portrayed as a doctor with poor bedside manner; the high-competence source was portrayed as a doctor who would at the end of the consultation receive a fellowship and the low-competence source was portrayed as a doctor who would instead be sued for malpractice (videos: DOI 10.6084/m9.figshare.7300088).

**Figure 1:**
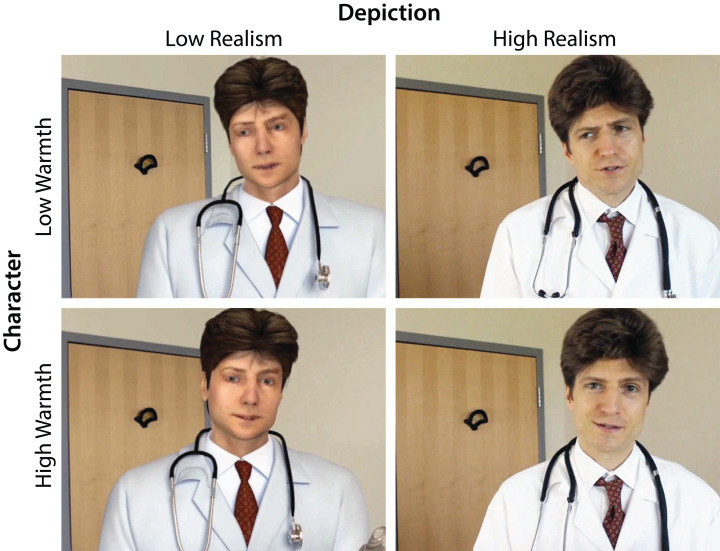
The real human actor and his digital double used in the experiment. In the role of a patient in a hypothetical virtual consultation, the participant interacted with a doctor. A 2 × 2 × 2 between-groups posttest-only experimental design was used. The doctor’s Character was good or poor bedside manner, Outcome was received a fellowship or sued for malpractice, and Depiction was a recorded video of a real human actor or of his 3D computer-animated double. Character, Outcome, and Depiction were designed to manipulate the doctor’s level of warmth, competence, and realism, respectively.

In research on how physical appearance influences persuasion, attractiveness has been the most widely investigated dimension. Attractiveness increases persuasiveness (Chaiken, 1979; Holzwarth, Janiszewski & Neumann, 2006; Jin & Bolebruch, 2009; Joseph, 1982; Khan & Sutcliffe, 2014; LaCrosse, 1975; Pallak, 1983; Pallak, Murroni & Koch, 1983), though with exceptions (Skalski & Tamborini, 2007). We would expect inconsistent realism and eeriness to have the opposite effect. Nevertheless, Patel & MacDorman (2015) found that although an animated character was more eerie, less attractive, and less human than its real counterpart, it did not reduce the intention to comply with advice. Based on the assumption that flaws in realism cause negative affective evaluations, namely, eeriness that decreases source credibility, we hypothesized that (H3) *a high-realism source will be more persuasive than a low-realism source.* In the virtual consultation, the high-realism source was the doctor depicted by a real human actor and the low-realism source was the doctor depicted by the actor’s computer-animated double (Fig. 1).

The future success of virtual consultations depends on patients’ willingness to accept this new technology. To this end, our study also aims to investigate the ways in which the message source’s warmth, competence, and realism influence the enjoyment of the virtual consultation. Smiling and other nonverbal cues indicating warmth and social presence have been found to increase enjoyment and trust when interacting with virtual human characters (Guadagno, Swinth & Blascovich, 2011; Qiu & Benbasat, 2009). Whether virtual or real, given that a doctor’s warmth and competence increase patient satisfaction (Kim, Kaplowitz & Johnston, 2004; Ohana & Mash, 2015; Willson & McNamara, 1982), we hypothesized that *the consultation will be more enjoyable* (H4) *with the high-warmth source than with the low-warmth source* and (H5) *with the high-competence source than with the low-competence source.*

Computer animation can often be more enjoyable than reality. For example, virtual human characters can increase satisfaction more than real people (Bickmore, Pfeifer & Paasche-Orlow, 2009). However, the characters tested were typically less realistic than the human model employed in this study and thus—according to Mori (2012) uncanny valley hypothesis—were less likely to appear eerie. Nevertheless, animation realism can increase a virtual character’s appeal and even uncanniness can elicit positive affect as exhibited by facial expressions and self-reports (Kokkinara & McDonnell, 2015; Mäkäräinen et al., 2015). Leaving open the direction of the effect, we hypothesized that (H6) *realism will influence the enjoyment of the virtual consultation.*

## Methods

The experiment was set up as a web-based interactive visual narrative. Participants playing as patient selected responses to a video of a dramatic character playing as doctor in a hypothetical virtual consultation. The doctor’s bedside manner, personal outcome, and depiction were manipulated to examine whether warmth, competence, and realism, respectively, increase adherence intention and enjoyment; and whether eeriness decreases adherence intention and enjoyment. Warmth was manipulated based on the scripted narrative through the doctor’s spoken words, voice and prosody, facial expressions, and body movements. Competence was manipulated through a subplot culminating in the doctor being either honored with a fellowship or sued for malpractice. Realism was manipulated by using a recorded video of either a real actor playing the doctor in a consultation room or computer-animated models simulating the same scenario.

### Participant characteristics and sampling

The sample was comprised of randomly selected undergraduate and graduate students, age 18 or older, from a Midwestern US public university system. Participation was voluntary, and testing was conducted at a time and location chosen by the participant.

The study was approved by the Indiana University Office of Research Administration (January 5, 2018, OHRP Category 7, Study No. 1712290464). Informed consent was obtained from all participants. Documentation of informed consent was waived under 45 CFR 46.117(c) or 21 CFR 56.109(c)(1). Explanation of aspects of the experiment that could have affected its outcome was delayed until after participation under 45 CFR 46.116(d). The research was performed in accordance with all relevant federal, state, and university standards, policies, and regulations on human subjects research.

### Research design

The experiment had a 2 × 2 × 2 between-groups posttest-only design (Independent variables). Each participant was randomly assigned to one of eight treatment groups, representing either a low or high level of warmth, competence, and realism.

### Procedure

After providing demographic information and reading introductory text, each participant assumed the role of a patient in a virtual consultation (Appendix A: Script). The consultation began with the patient’s blood sugar testing higher than normal. Dr. Richards appeared in a video wearing a white shirt, tie, and lab coat with a stethoscope draped over his shoulders. He was standing and holding a clipboard with the patient’s test results. The consultation proceeded through seven hypothetical doctor–patient exchanges and a final reply from the doctor.

In each doctor–patient exchange, the participant replied to the doctor by choosing one of four text-based responses. To maintain experimental control, the doctor’s statements were phrased to follow logically from any of the preceding responses. After the consultation, the participant completed a questionnaire for the posttest indices (Dependent variables).

### Independent variables

The independent variables were Character, Outcome, and Realism. Each variable had a high and a low level. These constituted the eight treatment conditions.

#### Character

Dr. Richards’s Character was reflected in his bedside manner, which was either good or poor. Character was represented by approximately 40% of the doctor’s dialogue. The good-manner treatments included expressions of caring, encouragement, praise, and confidence in the patient and treatment, offers of availability and support, and recommendations of external resources. The poor-manner treatments included complaining about others, using disparaging and offensive language, showing a lack of availability, demeaning and discouraging the patient, cracking a joke at the patient’s expense, and assuming the patient had petrifying fears and ingrained habits.

Dr. Richards’ remaining dialogue (337 of 553–561 words) was identical between the good-manner and poor-manner conditions. In this dialogue, Dr. Richards interpreted the patient’s test results as possibly indicating diabetes and invited the patient for a retest. He also explained type 1 and type 2 diabetes and their symptoms, complications, biological mechanism, and treatments.

#### Outcome

Dr. Richards’ Outcome is either a fellowship or a malpractice lawsuit. A high- and low-competence subplot was set up immediately before the virtual consultation. First, the participant overheard another patient in the waiting room discussing a malpractice lawsuit, and subsequently the nurse called the patient back and mentioned that the doctor is up for an award. These events were framed more or less favorably in the high or low competence versions, respectively. The subplot culminated at the end of the consultation. In the high-competence condition, Nurse Larsson announced, “Great news, Dr. Richards! The American College of Physicians is honoring you with a Fellowship!” In the low-competence condition, Nurse Larsson announced, “Bad news, Dr. Richards! Meredith Pratley decided to go ahead with the malpractice lawsuit.”

#### Depiction

The Depiction of the entire virtual consultation was either real or computer animated; however, both versions used the same recording, namely, the voice of the actor playing Dr. Richards and off-screen actress playing Nurse Larsson. The high-realism treatment used a recorded video of the actor (Fig. 1). The low-realism treatment used a video of a computer model developed from high-resolution reference photographs of the same actor. The actor’s clothing, props, and environment were all developed the same way. The computer models were animated manually using the real video as a reference. The animated Dr. Richards’ lips were synchronized to the real actor’s speech. The affective lip and eyebrow movements were precisely synchronized to the video by hand but appeared less emphatic. The same computer model had been used in a different scenario and found to be significantly more eerie than the real actor (Patel & MacDorman, 2015).

### Dependent variables

The semantic differential scales comprising the posttest indices are listed in Appendix B. The indices represent unweighted averages of interval measurements from their respective semantic differential scales. The scales were implemented as visual analogue scales (Funke & Reips, 2012; Reips & Funke, 2008). Each scale was represented by an adjective (or phrase) and its antonym on opposite ends of a horizontal bar. The participant had to place a mark on the bar, and depending on where the mark was placed, a decimal value between −1.000 and 1.000 was recorded for that scale. Likert scales were also implemented as visual analogue scales with *Strongly Disagree* and *Strongly Agree* on opposite ends of the horizontal bar. Index and scale order were randomized.

#### Warmth and competence

Dr. Richards’ warmth and competence were measured using three source credibility indices (McCroskey & Teven, 1999): Goodwill, Trustworthiness, and Competence. Goodwill and Trustworthiness formed a single Warmth index.

#### Human realism and eeriness

Dr. Richards’ human realism and eeriness were measured using three source appearance indices: Realism, Humanness, and Eeriness (Ho & MacDorman, 2017). Realism and Humanness formed a single Human Realism index.

#### Adherence intention

Intention to adhere to Dr. Richards’ treatment advice was measured using an index designed specifically for this virtual consultation.

#### Enjoyment

Narrative appreciation of the consultation was measured using an enjoyment index. To design the enjoyment index, the program evaluation index (Perry et al., 1997) was converted from intensity scales to semantic differential scales by adding antonyms.

## Results

### Participants

Overall, 738 participants randomly assigned to eight groups, with 88–105 in each group, completed the experiment (73% female, *n* = 538). As a manipulation check, a subset of 222 participants, with 21–34 in each group, completed additional items measuring source credibility and appearance (70.72% female, *n* = 157).

### Recruitment period and baseline

The experiment was conducted from January 15 to March 10, 2018. Most participants grew up in the US (88%, *n* = 647). They ranged in age from 18 to 82 (Mdn = 23, IQR = (20, 29)).

### Data analysis preliminaries

Test statistics were two tailed and interpreted at a 0.05 significance level. Partial eta squared (η_p_^2^) was interpreted with small = 0.01, medium = 0.06, and large = 0.14 thresholds (Cohen, 1973) and Cronbach’s α with acceptable = 0.7, good = 0.8, and excellent = 0.9 thresholds. Factor analysis used oblimin rotation and parallel analysis for the number of factors. All betas (β) were standardized. Correlations and path analysis of the structural models were performed in Lavaan 0.6, and other analyses in Jamovi 0.9.

### Index reliability

Goodwill and Trustworthiness scales loaded on the same factor and together comprised Warmth. Realism and Humanness scales loaded on the same factor and together comprised Human Realism. Three scales not contributing to reliability were removed, specifically, Without definite lifespan—Mortal was removed from Human Realism, Uninspiring—Spine-tingling was removed from Eeriness, and item 7 was removed from Adherence Intention (Appendix B). The revised Human Realism, Eeriness, and Adherence Intention indices were used in the analyses. All indices showed internal reliability (Table 1).

**Table 1:** Psychometric properties of the dependent variables.

DV	Items	*N*	*M*	SD	α	Skew
Warmth	12	222	−0.12	0.49	0.97	0.09
Competence	6	222	.19	0.44	0.93	–0.47
Human Realism	7	222	−0.28	0.38	0.86	0.28
Eeriness	7	222	−0.02	0.32	0.73	0.14
Adherence Intention	7	738	.08	0.43	0.87	–0.20
Enjoyment	12	738	−0.20	0.35	0.90	0.16

### Manipulation checks

As described below, the manipulation checks found that Warmth, Competence, and Human Realism varied as expected; however, Character also increased Competence and decreased Eeriness. Depiction had a nonsignificant effect on Eeriness, thus indicating that the Eeriness manipulation check was unsuccessful.

Using Pillai’s trace, a three-way MANOVA found a significant effect of Character, *V* = 0.54, *F*(4, 211) = 62.37, *p* < 0.001, and Depiction, *V* = 0.17, *F*(4, 211) = 11.18, *p* < 0.001, on the manipulation check variables. Separate univariate ANOVAs were conducted to test the main and interaction effects of Character, Outcome, and Depiction on Warmth, Competence, Human Realism, and Eeriness (Table 2). Character had a significant main effect on Warmth, Competence, and Eeriness with large effect sizes. Post hoc tests (Tukey’s HSD) showed that the good-manner doctor was rated significantly higher than the poor-manner doctor on Warmth and Competence and significantly lower on Eeriness. Character × Outcome interaction effect on Competence was significant with a small effect size, SS = 0.70, *F* = 4.63, *p* = 0.033, η_p_^2^ = 0.02.

**Table 2:** ANOVAs of Character, Outcome, and Depiction on Warmth, Competence, Human Realism, and Eeriness.

IV	DV	SS	*F*	*p*	η_p_^2^	*M*_diff_	SE	d*f*	*t*	*p*_tukey_
Character	Warmth	24.25	193.99	<0.001	0.48	0.67	0.05	214	13.9	<0.001
	Competence	7.83	52.10	<0.001	0.20	0.38	0.05	214	7.2	<0.001
	Human Realism	0.04	0.30	0.584						
	Eeriness	3.54	41.33	<0.001	0.16	–0.26	0.04	214	–6.4	<0.001
Outcome	Warmth	0.24	1.94	0.165						
	Competence	0.69	4.59	0.033	0.02	0.11	0.05	214	2.1	0.033
	Human Realism	0.02	0.13	0.724						
	Eeriness	0.00	0.01	0.917						
Depiction	Warmth	0.38	3.04	0.083						
	Competence	0.52	3.46	0.064						
	Human Realism	2.64	19.66	<0.001	0.08	–0.22	0.05	214	–4.4	<0.001
	Eeriness	0.01	0.17	0.680						

**Note:**

*N* = 222.

Outcome had a significant effect on Competence with a small effect size. The high-competence doctor (awarded fellowship) was rated significantly higher than the low-competence doctor (malpractice lawsuit) on Competence.

Depiction had a significant effect on Human Realism with a medium effect size but a nonsignificant effect on Eeriness. The high-realism doctor was rated higher than the low-realism on Human Realism.

### Regression analysis

Multiple linear regression was performed to predict Adherence Intention from Warmth, Competence, Human Realism, Eeriness, and Enjoyment. Although the first model was significant, *F*(5, 216) = 67.9, *p* < 0.001, *R*^2^ = 0.61, adj. *R*^2^ = 0.60, Enjoyment was nonsignificant, *t* = 1.52, *p* = 0.129, β = 0.08, and it was removed from the second model. Furthermore, the second model was significant, *F*(4, 217) = 83.7, *p* < 0.001, *R*^2^ = 0.61, adj. *R*^2^ = 0.60, but Human Realism was nonsignificant, *t* = −1.33, *p* = 0.192, β = −0.06, and it was removed from the third model, which had the best fit, *F*(3, 218) = 111.0, *p* < 0.001, *R*^2^ = 0.60, adj. *R*^2^ = 0.60 (Table 3). Warmth and Competence predicted Adherence Intention with medium-to-large effect sizes while Eeriness inversely predicted Adherence Intention with a small effect size.

**Table 3:** Regression model coefficients for Adherence Intention.

Predictor	*B*	SE	β	*t*	*p*
Intercept	0.12	0.02		4.77	<0.001
Warmth	0.40	0.06	0.48	6.69	<0.001
Competence	0.27	0.06	0.29	4.51	<0.001
Eeriness	−0.14	0.07	−0.11	–2.11	0.036

**Note:**

*N* = 222.

### Hypotheses testing

Using Pillai’s trace, a three-way MANOVA found a significant effect of Character, *V* = 0.28, *F*(2, 729) = 143.78, *p* < 0.001, Depiction, *V* = 0.02, *F*(2, 729) = 7.36, *p* < 0.001, and Outcome, *V* = 0.02, *F*(2, 729) = 6.40, *p* = 0.002 on dependent variables. Separate univariate ANOVAs were conducted to test the main and interaction effects of Character, Outcome, and Depiction on Adherence Intention and Enjoyment. All main effects were significant (Table 4). No significant interaction effects were found.

**Table 4:** ANOVAs of Character, Outcome, and Depiction on Adherence Intention and Enjoyment.

IV	DV	SS	*F*	*p*	η_p_^2^	*M*_diff_	SE	d*f*	*t*	*p*_tukey_
Character	Adherence Intention	37.14	282.86	<0.001	0.28	0.45	0.03	730	16.8	<0.001
	Enjoyment	1.94	16.59	<0.001	0.02	0.10	0.03	730	4.1	<0.001
Outcome	Adherence Intention	0.78	5.95	0.015	0.01	0.07	0.03	730	2.4	0.015
	Enjoyment	1.17	10.01	0.002	0.01	0.08	0.03	730	3.2	0.002
Depiction	Adherence Intention	1.07	8.19	0.004	0.01	0.08	0.03	730	2.9	0.004
	Enjoyment	1.30	11.06	<0.001	0.02	0.08	0.03	730	3.3	<0.001

**Note:**

*N* = 738.

**Figure 2:**
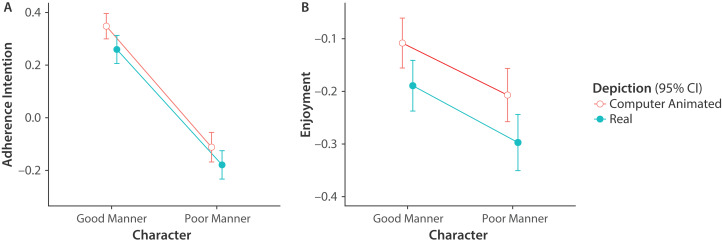
Means and 95% confidence intervals of Adherence Intention and Enjoyment plotted by Character and Depiction. Adherence Intention (A) and consultation Enjoyment (B) were significantly greater for the doctor with good bedside manner than the doctor with poor bedside manner.

H1 predicted that the high-warmth source will be more persuasive than the low-warmth source. The main effect of Character on Adherence Intention was significant and large. In the narrative, intention to adhere to the treatment advice of the doctor with good bedside manner was greater than intention to adhere to the treatment advice of the doctor with poor bedside manner (Fig. 2). Thus, H1 was supported.

H2 predicted that the high-competence source will be more persuasive than the low-competence source. The main effect of Outcome on Adherence Intention was significant but small. Intention to adhere to the treatment advice of the doctor awarded the fellowship was greater than intention to adhere to the treatment advice of the doctor sued for malpractice. Thus, H2 was supported.

H3 predicted that the high-realism source will be more persuasive than the low-realism source. It should be noted that the Realism manipulation check was successful, but the Eeriness manipulation check was not. The main effect of Depiction on Adherence Intention was significant but small. Intention to adhere to the advice of the computer-animated doctor was greater than intention to adhere to the advice of the doctor portrayed by a real human actor. Thus, H3 was not supported.

H4 predicted that the consultation will be more enjoyable with the high-warmth source than the low-warmth source. The main effect of Character on Enjoyment was significant but small. The consultation with the doctor with good bedside manner was more enjoyable. Thus, H4 was supported.

H5 predicted that consultation will be more enjoyable with the high-competence source than the low-competence source. The main effect of Outcome on Enjoyment was significant but small. The consultation was more enjoyable with the doctor awarded the fellowship. Thus, H5 was supported.

H6 predicted that realism will influence enjoyment of the virtual consultation. The main effect of Depiction on Enjoyment was significant but small. The consultation with the computer-animated doctor was more enjoyable. Thus, H6 was supported.

### Secondary analysis

Depiction (high realism) was predicted to reduce Eeriness; however, the results revealed that Depiction actually reduced Enjoyment and that Character (good manner) reduced Eeriness. Surprisingly, Depiction and Eeriness were uncorrelated (Table 5).

**Table 5:** Correlations for analysis of path models of Warmth.

Variables	Warmth	Eeriness	Enjoyment	Human Realism	Depiction
Warmth	—				
Eeriness	−0.55***	—			
Enjoyment	0.48***	−0.19**	—		
Human Realism	0.29***	−0.31***	0.47***	—	
Depiction	−0.14*	0.00	−0.22**	0.29***	—

**Notes:**

*N* = 222.

* *p* < 0.05.

** *p* < 0.01.

*** *p* < 0.001.

To explain this, we proposed that enjoyment of the consultation with the computer-animated Dr. Richards interfered with the measurement of Eeriness. However, this interference appeared to be indirect because, although Enjoyment and Eeriness were significantly correlated, only Human Realism and Warmth were significant predictors of Eeriness in a regression model. Similarly, only Eeriness and Enjoyment were significant predictors of Warmth; only Warmth, Human Realism, and Depiction were significant predictors of Enjoyment; and only Enjoyment, Eeriness, and Depiction were significant predictors of Human Realism. Each of these four regression models can be represented by a causal directed graph with an arrow from each predictor variable to the outcome variable. Each of these four graphs could contribute variables and edges to a larger directed graph. Thus, the variables and edges of these graphs identified the potential set of path models we sought to analyze by structural equation modeling.

A direct effect, represented by an arrow, is a hypothesized relation between two variables. The strength of this relation is a free parameter, which is estimated during model identification. The absence of an arrow between two variables is a fixed parameter (fixed to 0). A nonsignificant *p-*value of a free parameter indicates poor local fit. An upper bound on the number of free parameters was set at 11 by applying the *N:q* rule, which recommends a minimum 20:1 ratio of sample size to free parameters (Jackson, 2003).

By the parsimony principle, we specified the simplest model with the highest priority effect first and then proceeded to specify more complex models as necessary by adding direct effects (Kline, 2016). The simplest model, Model 0, had a direct effect from the independent variable Depiction to Eeriness. This model was rejected because the local fit was nonsignificant (β = 0.00, *p* = 0.966).

**Figure 3:**
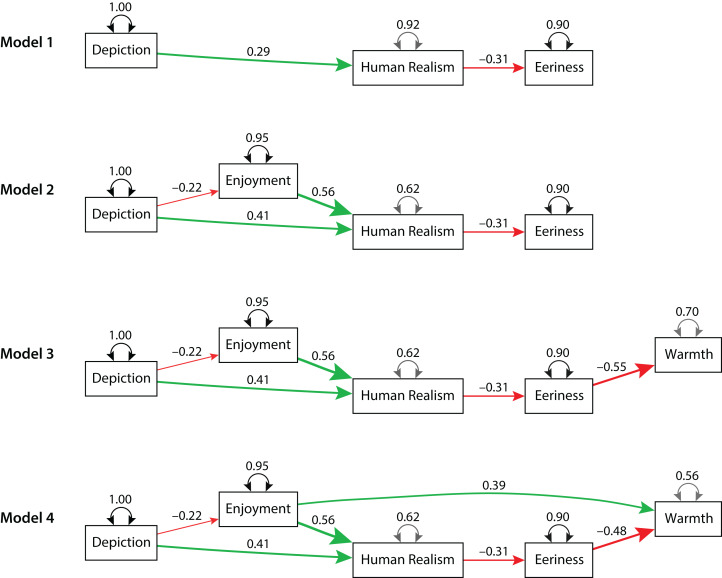
Path models of the indirect effect of Enjoyment of computer animation on Eeriness. In the path models, the green arrow indicates a positive direct effect, the red arrow indicates a negative direct effect, and the standardized estimate (β) indicates the strength of the effect. The independent variable Depiction, indicating computer animated or real, is the exogenous variable. In Model 2, 3, and 4, Enjoyment mitigated Eeriness by increasing Human Realism. All models had good local fit, but only Model 2 and 4 had good global fit (Table 6). Model 4 is the final model.

In Model 1, Depiction had a direct effect on Human Realism, and Human Realism had a direct effect on Eeriness. Model 1 is shown in Fig. 3. The green arrow indicates a positive direct effect, the red arrow indicates a negative direct effect, and the width of each arrow indicates the magnitude of the direct effect. The strength of the direct effects is indicated by the standardized estimate (β) next to the green arrow and the red arrow. All estimates were significant (*p*s ≤ 0.001), and all of the following criteria for acceptable global fit were met: *p* > 0.05 (cannot reject the exact fit hypothesis), RMSEA ≤ 0.08 (a cutoff for marginal fit; MacCallum, Browne & Sugawara, 1996), RMSEA $\hat \varepsilon_{\rm{L}}$ = 0 (confidence interval includes zero), and the combination rule (CFI ≥ 0.95 and SRMR ≤ 0.08; Hu & Bentler, 1999). However, Model 1 excludes the theoretically important variable Enjoyment. Table 6 lists the global fit statistics for this and subsequent path models.

**Table 6:** Global fit statistics for path models of Eeriness (1, 2) and of Eeriness and Warmth (3, 4, 4a, 4b).

Model	Model χ^2^			*q*	RMSEA		CFI	SRMR
	χ_M_^2^	d*f*_M_	*p*		$\hat \varepsilon $	90% CI		
1	2.357	1	0.125	7	0.078	[0.000, 0.213]	0.967	0.038
2	2.393	2	0.302	6	0.030	[0.000, 0.140]	0.997	0.033
3	56.812	5	0.000	9	0.216	[0.168, 0.268]	0.807	0.122
4	4.162	4	0.385	10	0.014	[0.000, 0.103]	0.999	0.040
4a	12.316	4	0.015	10	0.097	[0.038, 0.161]	0.969	0.039
4b	12.316	4	0.015	10	0.097	[0.038, 0.161]	0.969	0.039

**Note:**

*q*, number of free parameters; CI, confidence interval.

For Model 2, all estimates were significant (*p*s ≤ 0.001), and all global fit criteria were met. However, Model 2 excludes the theoretically important variable Warmth. Model 3 added a direct effect from Eeriness to Warmth (β = −0.55, *p* < 0.001). Although estimates were significant (*p*s ≤ 0.001), none of the global fit criteria were met. Thus, Model 3 was rejected. Model 4 added a direct effect from Enjoyment to Warmth (β = 0.39, *p* < 0.001). All estimates were significant (*p*s ≤ 0.001), and all global fit criteria were met. Thus, Model 4 was accepted. It is our final model.

As a best practice, it is recommended to compare the final model with theoretically plausible alternative models (Kline, 2016). Model 4 was compared with two alternative models by switching the direction of the direct effect between Eeriness and Warmth (Model 4a) and between both Eeriness and Warmth and Enjoyment and Human Realism (Model 4b). Although all estimates were significant (*p*s ≤ 0.003) for these models, Model χ^2^ and RMSEA global fit criteria were unmet. Thus, these alternative models were rejected.

### Data availability

All datasets and R scripts for the analyses are available as Supplemental Material.

## Discussion

Each year, nonadherence costs the US healthcare system more than $300 billion and results in 250,000 deaths (Balkrishnan, 2005; Benjamin, 2012). The global human and financial costs are far greater (Cutler et al., 2018). Real clinicians are a limited resource (Chang et al., 2012; Pickersgill, 2001; Sklar, 2013) as many countries are facing a shortage of physicians, nurses, and other healthcare professionals (Bodenheimer, Chen & Bennett, 2009; Bodenheimer & Smith, 2013; Colwill, Cultice & Kruse, 2008; Potempa, Redman & Landstrom, 2009). These shortages reduce opportunities for the clinical supervision of patients, allowing their nonadherence to increase negative health outcomes. Developing virtual consultations to promote adherence could improve patient health while reducing healthcare costs and usage (Roebuck et al., 2011).

Virtual consultations that use real-time computer animation have many advantages over films and other more traditional interventions. Animated virtual consultations can be revised quickly for immediate distribution on the Internet (e.g., using software for real-time animation or video capture in a game engine). Without hiring actors, virtual clinicians can be adapted to different conditions and treatments, internationalized and localized to different languages and cultures, and tailored to the patient’s demographic group (DeSmet et al., 2014). The controllability of virtual humans and reproducibility of their behavior make virtual consultations an effective platform for experiments investigating clinical interactions. Because virtual humans can assume the patient’s role, they can also be used to teach and assess clinical and communication skills (Cook & Triola, 2009; Parsons et al., 2008; Persky, 2011; Persky & Eccleston, 2011). Thus, using computer-animated healthcare providers in clinical settings offers a promising, cost-effective approach to increase patients’ health literacy and adherence to treatment advice (Bickmore, Pfeifer & Jack, 2009; Bickmore et al., 2010b).

As predicted, good bedside manner and a subplot highlighting the doctor’s competence increased intention to adhere to treatment advice among role-playing participants in a hypothetical virtual consultation. Surprisingly, adherence intention was lower for the doctor portrayed by a real human actor than by his digital double. Thus, the uncanny valley phenomenon posed no threat to the effectiveness of the intervention. On the contrary, the computer-animated doctor increased adherence intention and consultation enjoyment significantly more than the real human. Contrary to our previous study, the same digital double used in Patel & MacDorman (2015) was no more eerie than the same human actor, although eeriness predicted lower warmth. We believe that enjoyment of the animation caused the doctor to be perceived as warmer and more human, which mitigated the negative effects of eeriness associated with the uncanny valley. This explanation is compatible with a path model derived from the data. Notably, poor bedside manner increased eeriness.

### Limitations and future work

The results identified two potential threats to internal validity owing to the methodology. First, the measurement of eeriness was affected by character traits, such as warmth and competence. For example, the doctor with poor bedside manner was rated significantly eerier than the doctor with good bedside manner. The eeriness index was designed to measure eeriness caused by the uncanny valley, which is typically exhibited by inconsistencies in human realism, and not by a cold personality (Ho & MacDorman, 2010). The same computer-animated model that was rated eerier than its human counterpart in Patel & MacDorman (2015) was not significantly different in the present study. This may indicate how cues about character traits can dilute an index intended to measure eeriness (Zibrek, Kokkinara & McDonnell, 2018). To control for the compounding effects of the doctor’s bedside manner and outcome on eeriness, the experiment could administer eeriness scales in a pretest: participants would rate the doctor portrayed by either a real human actor or his digital double in a neutral setting—without any cues on warmth, coldness, competence, or incompetence, and before the narrative is introduced.

A second threat to internal validity concerns difficulty in separating computer animation’s positive contribution to adherence intention and consultation enjoyment from the uncanny valley’s possible negative contribution. This can be achieved by employing two versions of the computer-animated doctor, one with greater inconsistency in human realism than the other. Thus, a follow-up experiment could control for the compounding effects of computer animation on enjoyment by independently varying aspect aspects of animation found to modulate eeriness.

A threat to external validity is the use of role-playing participants instead of participants who fit the scenario, namely, patients in the initial stage of being diagnosed with type 2 diabetes. A number of studies suggest our results may generalize to actual patients. Baylor et al. have found animated pedagogical agents to be effective in increasing motivation and learning (Baylor, 2009, 2011; Baylor & Kim, 2005; Baylor & Ryu, 2003; Kim, Baylor & Shen, 2007; Plant et al., 2009). Reviews of the literature have found interactive media with immersive narratives to be effective in promoting behavior change and in improving health outcomes (Baranowski et al., 2008; DeSmet et al., 2014; Lu et al., 2012; Read & Shortell, 2011; Thompson, 2012). Interpreting the results of the present study in light of these findings, we believe a computer-animated doctor could be effective in clinical settings. Therefore, to confirm effectiveness, a follow-up experiment should be conducted with clinical patients. However, substantial changes to the script would be required to ensure patients assigned to the low warmth and low competence conditions were not harmed relative to current best practices. In addition, certain dramatic elements of the script would need to be revised to render it medically accurate and ethical to employ in a clinical setting.

## Conclusions

The uncanny valley is the experience of perceiving simulated humans as eerie (Mangan, 2015). Mori (2012), who proposed the theory in 1970, attributed the phenomenon to some features of the simulation appearing more human than others. Several empirical studies support his claim (Chattopadhyay & MacDorman, 2016; MacDorman et al., 2009; MacDorman & Chattopadhyay, 2016; Mitchell et al., 2011; Seyama & Nagayama, 2007). This study examined the uncanny valley in a hypothetical virtual consultation, casting in the role of doctor either a real human actor or his digital double.

With the exception of Patel & MacDorman (2015), previous experiments on how human realism affects persuasion have not considered the uncanny valley. Further, they have lacked the experimental control of using a digital double to simulate the appearance and behavior of a real human. No previous study has addressed these issues with respect to a patient’s intention to adhere to treatment advice.

The findings show the effectiveness of a digital double relative to a real human in promoting adherence intention with role-playing participants. In addition, they show increased enjoyment of the consultation with the computer-animated doctor, which could indicate future acceptance of the technology. Cues of the doctor’s warmth and competence remained effective despite the use of realistic computer animation. Thus, insofar as the uncanny valley may have come into play, it did not impede the effectiveness of the computer-animated virtual consultation.

Patient nonadherence to treatment poses a major threat to public health (Iuga & McGuire, 2014; Vermeire et al., 2001). Our findings suggest that an animated virtual consultation could be more effective and enjoyable than a consultation filmed with a real human actor. Further testing is needed in clinical settings. The effectiveness of the animated virtual consultation with role-playing participants should encourage the development and clinical testing of this technology, which has advantages over creating content with human actors, such as ease of scenario revision, internationalization, localization, personalization, and web distribution (Bickmore, Pfeifer & Jack, 2009; Bickmore, Pfeifer & Paasche-Orlow, 2009; Bickmore et al., 2010a; DeSmet et al., 2014).

## Appendix A: Script

[Text introducing the narrative].

Outcome Treatment 1

*Both conditions:* One afternoon, you visit Dr. Richards, a primary care physician, for a routine physical examination. As instructed by the nurse, Jane Larsson, you ate nothing on the morning of your appointment.

*Low Competence:* In the waiting room, two women are seated together, and you hear one of them say, “Meredith, you deserve compensation. You have a strong case.”

*High Competence:* In the waiting room, you overhear a patient, Meredith Pratley, tell her husband, “The lawyer says, if we sue the doctor, we both could make a ton of money.”

*Both conditions:* After the examination, you learn from Dr. Richards that your blood sugar tested higher than normal. You undergo some further blood work and return to the waiting room. After a while, Nurse Larson calls you back into the doctor’s office.

*Low Competence:* Walking to the office, the nurse whispers, “Dr. Richards is up for an award. I’m sure it will go to his head!”

*High Competence:* Walking to the office, the nurse gushes, “Dr. Richards is up for an award. Today we find out the result!”

You are greeted by Dr. Richards.

[*Depiction Treatment:* The virtual consultation begins with either computer animation or a recorded video].

Character Treatment 1

Dr. Richards, *Poor Manner:* [Rolling eyes peevishly and shaking head]. How’d you like our noisy waiting room? It sounds like the ghetto clinic where Dr. Mehta volunteers. And they’re all here for *me*. I inherited this disaster when she took maternity leave so…

Dr. Richards, *both conditions:* I’m sorry you were kept waiting. From your latest blood work, your fasting blood sugar level is 203, which is higher than we’d like to see. If it tests above 126 again, we’ll have to consider the possibility that you have diabetes.

Dr. Richards, *Good Manner:* But don’t be too concerned. It’s not clear you have it, and even if you do, it’s very common, and we know how to treat it.

[Participant selects a response].

- What is the chance I have diabetes?
- When will you know for sure whether I have diabetes?
- Will you retest my blood glucose level today?
- Do my other lab results also indicate diabetes?

Character Treatment 2

Dr. Richards, *Good Manner:* Again, I wouldn’t be too concerned because although…

Dr. Richards, *both conditions:* Your hemoglobin A1C and triglycerides are elevated. These might be signs of diabetes or a prediabetic condition. To be sure, we’ll need to retest you. Come back at least 12 hours after taking a meal without processed sugar. I want you to get the results fast, so I’ll fit you in tomorrow…

Dr. Richards, *Poor Manner:* If somebody cancels. Right now, I have to reserve my one open slot for a true emergency.

[Participant selects a response].

- What should I know about diabetes?
- What type of diabetes do I have?
- Is it more likely I have type 1 or type 2 diabetes?
- What is the difference between type 1 and type 2 diabetes?

Character Treatment 3

Dr. Richards, *Poor Manner:* You should know [chuckling] it doesn’t really matter what type of diabetes you have because…

Dr. Richards, *both conditions:* Diabetes, both type 1 and 2, involves your blood sugar being high enough to put your health at risk. This is due to a lack of insulin. In type 1 your body doesn’t make any insulin, because the cells that make it in the pancreas have been killed by the immune system. In type 2 your body makes some insulin, but it’s either not enough or not used well. For both types, the most common symptoms are excessive thirst and frequent urination. If it turned out you had diabetes, it’s probably type 2, because of the late onset.

Dr. Richards, *Good Manner:* I certainly hope you have neither type, but type 2 is less severe and easier for us to treat.

[Participant selects a response].

- I don’t feel thirsty that often.
- I don’t urinate very frequently.
- Do you have to feel thirsty and urinate frequently to have diabetes?
- I only have one of those symptoms.

Character Treatment 4

Dr. Richards, *Poor Manner:* [Incredulous]. If you’re like most patients, you don’t know how to assess your own symptoms and…

Dr. Richards, *both conditions:* It’s possible to show *no* symptoms. A third of those with diabetes don’t even know they have it. But it’s important to get treated to keep the symptoms from developing and to prevent complications.

Dr. Richards, *Good Manner:* I’m glad to say there are many treatments today that didn’t exist 20 years ago. Our understanding of the condition is constantly improving.

[Participant selects a response].

- What complications are caused by diabetes?
- What can I do to prevent complications related to diabetes?
- How does the disease progress?
- Do the complications of type 2 diabetes differ from type 1?

Character Treatment 5

Dr. Richards, *Poor Manner:* [Checks time on watch and scoffs] Look…

Dr. Richards, *both conditions:* The same complications are associated with both types: heart disease; kidney disease; blindness; a shortened lifespan. However, their risk can be greatly reduced with the right treatment.

Dr. Richards, *Good Manner:* So, don’t worry. If you have any health issues, we’ll do [emphatically] *everything* we can to help you.

[Participant selects a response].

- How does diabetes cause blindness?
- Why would high blood sugar shorten my life?
- How is a lack of insulin related to heart disease?
- How can diabetes lead to kidney disease?

Character Treatment 6

Dr. Richards, *Good Manner:* That condition might happen, far in the future, to someone who isn’t doing enough to control the disease. [Upbeat tone]. But you’re asking great questions.

Dr. Richards, *both conditions:* [Rising tone]. How does diabetes cause that? All your organ systems have one thing in common: they rely on proteins to function. If your body doesn’t make enough insulin, sugar binds to those proteins and keep them from working. This is why some people need to inject themselves with insulin.

Dr. Richards, *Poor Manner:* Have you ever made *crème brûlée?* Take some custard—your proteins—sprinkle on sugar and caramelize it with body heat. [Chuckling] You’re making *crème brûlée* out of your body.

[Participant selects a response].

- Earlier you mentioned treatment options.
- Could you tell me more about what I can do to improve my prospects?
- Will I have to inject myself with insulin?
- I don’t feel comfortable using needles.

Character Treatment 7

Dr. Richards, *Poor Manner:* [Nodding teasingly]. I bet you’re terrified of needles, but in the early stages of diabetes…

Dr. Richards, *both conditions:* Injections aren’t always necessary. Sometimes pills are enough. Getting plenty of exercise and eating a sensible diet are also key in stopping the disease from getting worse. We can discuss lifestyle changes at your next appointment…

Dr. Richards, *Poor Manner:* [Impatiently]…because I’ve got a waiting room full of people. You see, the trouble is your diet and exercise habits are so ingrained they’re almost impossible for you to change. So you might as well go straight to the pills or needles.

[Participant selects a response].

- What’s wrong with my current diet?
- What kind of diet and exercise program do you recommend?
- I feel I am already exercising enough.
- Is there a type of exercise that is best for people with diabetes?

Character Treatment 8

Dr. Richards, *Poor Manner:* Let’s stop here. The first time you hear [pause] *diabetes*, you freeze. That’s all you hear. You’re thinking, “I’m going to lose a foot! I’ll go blind!” So there’s really no point in talking about your case now. Take this brochure. Read it before your next appointment, and you’ll be able to ask better-informed questions.

Dr. Richards, *Good Manner:* It’s important to quantify and monitor improvements in diet and exercise, because people tend to underestimate their food intake and overestimate their exercise time. Working with a nutritionist and personal trainer can help with this. You might also consider joining a patient support group. My patients have told me they have learned more in a couple of weeks at a patient support group than in six months of coming to this office, because these groups are run by people who really know what it’s like to have the disease. Feel free to contact me if you have any questions or concerns. I look forward to seeing you tomorrow.

[Subplot ending].

Outcome Treatment 2

Low Competence:

Nurse Larsson: “Bad news, Dr. Richards! Meredith Pratley decided to go ahead with the malpractice lawsuit.”

Dr. Richards: “It’s unfortunate she feels that way.”

High Competence:

Nurse Larsson: “Great news, Dr. Richards! The American College of Physicians is honoring you with a Fellowship.”

Dr. Richards: “That *is* great news!”

[Virtual consultation ends].

## Appendix B: Indices

Indicate your level of agreement with the following statements about Dr. Richards.

*Goodwill*

1. Insensitive—Sensitive
2. Cares about me—Doesn’t care about me ^R^
3. Concerned with me—Not concerned with me ^R^
4. Not understanding—Understanding
5. Has my interests at heart—Doesn’t have my interests at heart ^R^
6. Self-centered—Not self-centered

*Trustworthiness*

1. Honorable—Dishonorable ^R^
2. Moral—Immoral ^R^
3. Untrustworthy—Trustworthy
4. Honest—Dishonest ^R^
5. Unethical—Ethical
6. Phony—Genuine

*Competence*

1. Intelligent—Unintelligent ^R^
2. Bright—Stupid ^R^
3. Inexpert—Expert
4. Incompetent—Competent
5. Informed—Uninformed ^R^
6. Untrained—Trained

*Realism*

1. Computer animated—Real
2. Replica—Original
3. Digitally copied—Authentic

*Humanness*

1. Inanimate—Living
2. Synthetic—Natural
3. Mechanical movement—Biological movement
4. Human-made—Humanlike
5. Without definite lifespan—Mortal

*Eeriness*

1. Ordinary—Creepy
2. Plain—Weird
3. Predictable—Eerie
4. Typical—Supernatural
5. Bland—Uncanny
6. Dull—Freaky
7. Boring—Shocking
8. Uninspiring—Spine-tingling

Adherence *Intention*

Indicate your level of agreement in your role as patient during the consultation.

Strongly Disagree—Strongly Agree

1. I’d return to Dr. Richards for diabetes retesting.
2. If I were diagnosed as prediabetic, I would consult a different doctor instead of Dr. Richards. ^R^
3. I’d ignore any treatment advice from Dr. Richards. ^R^
4. I’d double my number of meals and cut the quantity in half, if Dr. Richards said it would prevent complications.
5. I’d inject myself with insulin 30 minutes before each meal if Dr. Richards recommended it.
6. I’d follow Dr. Richards’ recommendation to exercise at least half an hour each day.
7. I could not rid my diet of sugar even if Dr. Richards ordered it. ^R^
8. I’d make any lifestyle change Dr. Richards’ suggests to stop the disease from progressing.

*Enjoyment*

1. Exciting—Ordinary
2. Suspenseful—Predictable
3. Boring—Interesting ^R^
4. Entertaining—Dull
5. Amusing—Tedious
6. Unimaginative—Imaginative ^R^
7. Depressing—Cheerful ^R^
8. Enjoyable—Unpleasant
9. Fun—Tiresome
10. Awkward—Adept ^R^
11. Professional—Amateurish
12. Humorous—Solemn

**Supplementary Material: Videos**

The videos may be viewed at DOI 10.6084/m9.figshare.7300088.

## Supplemental Information

10.7717/peerj-cs.168/supp-1 Supplemental Information 1 Dataset for hypothesis testing.

10.7717/peerj-cs.168/supp-2 Supplemental Information 2 Dataset for manipulation checks, regression, correlation, and path models.

10.7717/peerj-cs.168/supp-3 Supplemental Information 3 Dataset which allows the reproduction of all the results in the article including the figures and tables.

## Additional Information and Declarations

### Data Availability

The following information was supplied regarding data availability: The raw data can be found in the Supplemental Information.
